# Anisotropy parameters from shapes of ion-ion correlation features of fragmenting molecules

**DOI:** 10.1038/s41598-024-80014-z

**Published:** 2024-11-20

**Authors:** Emelie Olsson, Måns Wallner, Richard J. Squibb, Veronica Ideböhn, Marco Parriani, Michael A. Parkes, Stephen D. Price, John H.D. Eland, Raimund Feifel

**Affiliations:** 1 Department of Physics, University of Gothenburg, Origovägen 6B, Gothenburg, 412 58 Sweden; 2 Department of Civil and Environmental Engineering, University of Perugia, Via G. Duranti 93, Perugia, 06125 Italy; 3 Department of Chemistry, University College London, 20 Gordon Street, London, WC1H 0AJ UK; 4 Department of Chemistry, Physical and Theoretical Chemistry Laboratory, Oxford University, South Parks Road, Oxford, OX1 3QZ UK

**Keywords:** Atomic and molecular interactions with photons, Chemical physics

## Abstract

When a molecule loses two electrons, Coulomb repulsion makes the resulting doubly charged system likely to fragment into two singly charged ions. These monocations can be detected in a correlated fashion using multiplex time-of-flight spectroscopy. The island shapes in the ion-ion coincidence maps derived from such two-body dissociations contain detailed information on the physical processes underlying the fragmentation. Here, a simple method is presented where a fit function is used to determine the anisotropy parameter *β* of the molecular distribution from the peak shape of the time-of-flight difference of the two ions. The validity of the method is demonstrated by performing fits to simulated peak shapes, recovering the *β* value of the input angular distribution, and by comparison of experimental peak shapes to *β* values known from the literature.

**Supplementary Information:**

The online version contains supplementary material available at 10.1038/s41598-024-80014-z.

## Introduction

Investigations of the formation and dissociation of doubly charged positive ions using ion-ion coincidence time-of-flight (TOF) spectroscopy are long established and widespread (e.g. Ref.^1^ and refs. therein). The two-dimensional islands displayed in the coincidence data from two-body dissociations have previously been used to determine angular distributions and anisotropy parameters, without the use of position-sensitive detectors. This method requires the measurements to be repeated for different angles between the linearly polarized light and the detection axis (see e.g. Refs.^2,3^). One specific angle, the “pseudo-magic” angle of 54.7°, is used to determine the kinetic energy distributions of the fragments and instrumental parameters. Then, the anisotropy parameter is found by comparing the ion distributions for the two orthogonal polarization angles to simulated spectral profiles of the fragment ions with the previously established kinetic energy distributions. Dedicated angle-resolving photoionyield spectroscopy (ARPIS) set-ups, where fragment ions are concurrently detected in the axes parallel and perpendicular to the electric field vector of the incident light, can in addition to measuring the anisotropy reveal the symmetry of the excited states and provide information on bending motions in the excited states (e.g. Refs.^4,5^ and refs. therein). Alternatively, if a momentum imaging technique is used, the kinetic energy distribution is given directly from the measurement, and the anisotropy parameter is determined by comparison to simulations^6,7^.

In the present work, we demonstrate the use of a simple function for extracting the anisoptropy parameter *β* from the photoion-photoion coincidence (PIPICO) time-of-flight data of the positively charged species produced in the two-body dissociation detected only along a single axis with a one-dimensional detector, without the need for measurements at several different angles. The basic idea relies on the concept of time-focusing in TOF mass spectrometry which was originally introduced by Wiley and McLaren^8^. Under time focusing conditions, the central flight time of each ion species is independent of its initial position in the interaction region, and any deviations from the central flight time will be linearly proportional to the on-axis component of its initial momentum. For two-body dissociations of doubly charged molecules into monocations, the kinetic energy release (KER) gives the ions initial momenta that are typically much larger than their momenta from the thermal velocity of the neutral molecule in a two-body dissociation. Conservation of momentum ensures that the two fragment ions have equal and opposite linear momenta. Then, the resulting coincidence “island” feature in a two-dimensional heat map of intensity as a function of the flight times *t*_1_ and *t*_2_ of the two ions, is a simple bar with the slope of − 1 (unit negative gradient) with a length proportional to the square root of the largest value of KER. The intensity distribution *across* the bar, or equivalently the distribution of *t*_2_ + *t*_1_, shows the thermal velocity distribution of the parent molecule, convoluted with any resolution limitation of the apparatus. The shape of this distribution should ideally be Gaussian reflecting a one-dimensional Maxwell-Boltzmann distribution, giving the effective kinetic temperature of the target gas. If the target gas is supplied as a jet or molecular beam, the effective kinetic temperature transverse to the jet should be below ambient. The intensity distribution *along* the line, or equivalently the distribution of *t*_2_ − *t*_1_, i.e. the PIPICO peak shape, reflects the ion angular distribution in relation to the axis of the initial doubly-ionized molecules and also the distribution of KERs.

It is often assumed that the angular distribution of nascent doubly ionized molecules is isotropic in the laboratory frame. This assumption is partially justified in the case of direct double photoionization and in electron-impact double ionization. If such a distribution holds, each single-valued KER will give rise to a flat-topped peak shape in *t*_2_ − *t*_1_ with vertical ends (a “top hat”), of width proportional to the square root of the KER. Observed PIPICO peak shapes are the sum of all such rectangular shapes, derived from the kinetic energy release distribution (KERD). We note that, because of the (KER)^1/2^ dependence on the PIPICO width, it is difficult to extract exact KERDs from PIPICO peak shapes, and such extraction is typically better achieved by other techniques, such as those using position-sensitive detectors to determine the full three-dimensional momentum distribution. In most cases, the width of the KERD in charge separation is considerably smaller than the central magnitude of the KER, so rather sharp-ended PIPICO peak shapes are found. It is a customary approximation to derive a value for the mean KER from the full width of the peak at half height, but this can be done more accurately by using the variance of the peak^9,10^.

In what follows, we will show how angular information can be extracted from a single measurement using a Wiley and McLaren based apparatus instead of using either specialist angular-resolving apparatus or repeated measurements at different angles between the linearly polarized light and the detection axis.

## Results and discussion

In the following section, the anisotropy parameter *β* is found from PIPICO peak shapes using a fit function. The *β* parameter was introduced by Zare^11^ and is explained in more detail below in the “Theoretical model” section. The fit function is a convolution of *I*(*x*), which describes the projection of the three-dimensional anisotropic distribution of dissociated ions onto the time of flight axis, and a Gaussian *f*(*x*) with standard deviation *σ*, to account for all of the apparatus effects and the KER. Both *β* and *σ* are fit parameters, and the fit procedure is described in more detail below in the “Methods” section. To evaluate the fit function it is tested on simulated data and experimental data separately.

### Fit to simulated data

Simulations of two-body dissociations under conditions replicating our experimental setup were performed, described in the “Simulations” section below. First, the *t*_2_ − *t*_1_ distributions from the simulations were validated by comparison to experimental *t*_2_ − *t*_1_ distributions. Then, simulations of the dissociation of the dication OCS^2+^ to the monocation pairs OC^+^ + S^+^ and O^+^ + CS^+^ were performed, with different values for the anisotropy parameter *β* that describes the input distribution of the ions. The *β* parameter was varied in steps of 0.25 between − 1 and 2, for both linearly polarized light with polarization direction h*ν*_∥_ and h*ν*_⊥_. From each *t*_2_ − *t*_1_ distribution, the fit parameter *β* was found using the fit function described in the “Methods” section, and compared to the input *β* value. Typically the 95% confidence interval for *β* from fits to simulated data is less than ± 0.1. The contribution from the Gaussian apparatus width and KER distribution is small in the simulated data, possibly from a narrower KERD and apparatus broadening compared to the PIPICO shapes from experimental data. One example of a simulated PIPICO peak shape can be found in the Supplementary Fig. S1. We note that the simulated data is not used for further comparison to experimental data, but only to validate the use of the fit function on PIPICO peak shapes for values of *β* between − 1 and 2.

**Fig. 1:**
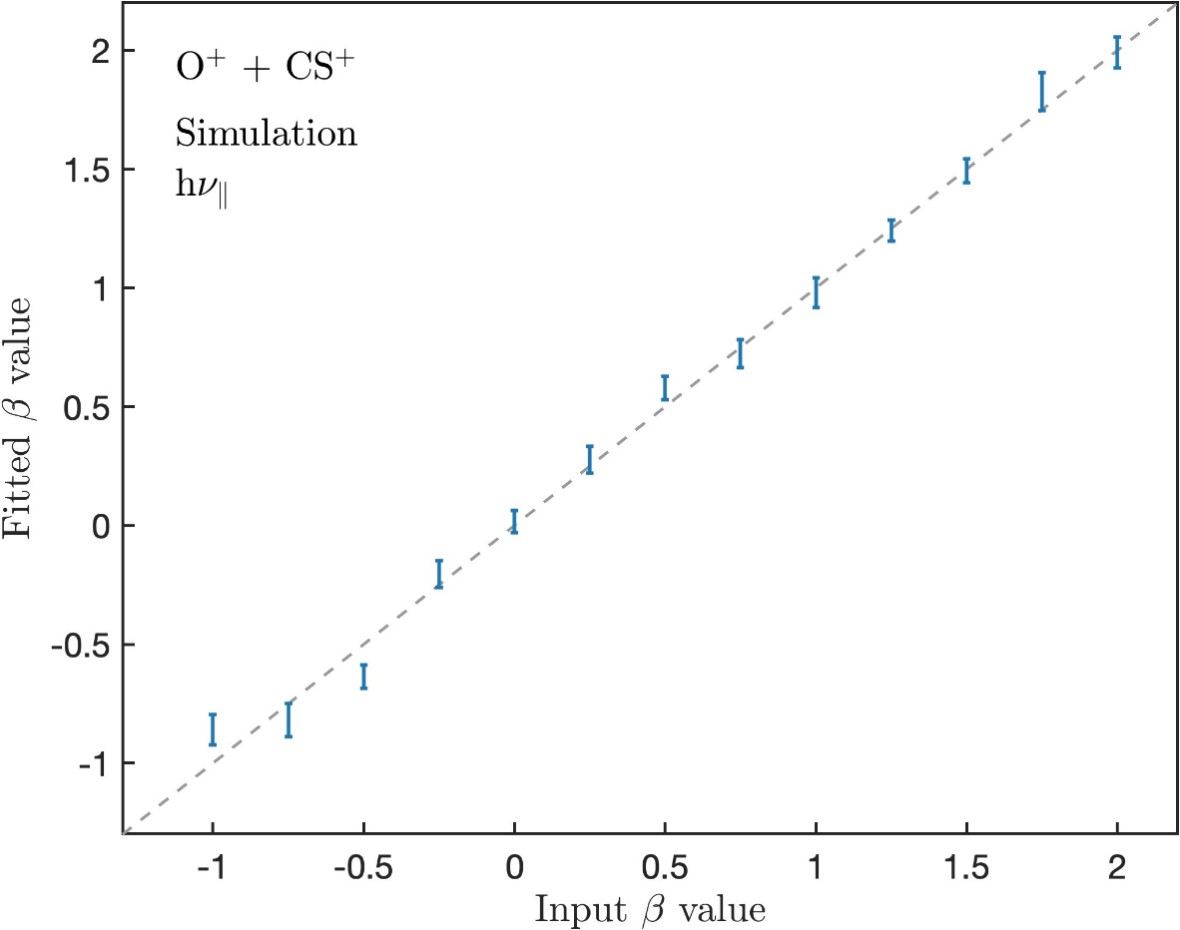
Comparison of the simulation input *β* and fitted *β* values using the simulation output for the O^+^ + CS^+^ dissociation, for h*ν*_∥_, where the error bars represent 95% confidence interval for *β*. The dashed line x = y is included as a guide for the eye.

A comparison between the input *β* value, describing the initial distribution of the ions in the simulation, and the *β* values from the fit to simulated data is shown in Fig. 1, for the O^+^ + CS^+^ dissociation and with the light polarization parallel to the flight tube axis (h*ν*_∥_). The error bar of each data point corresponds to the 95% confidence interval for *β*. Errors for the other two fit parameters are not represented in this figure. The corresponding data for the other polarization (h*ν*_⊥_) in addition to the OC^+^ + S^+^ dissociation similarly show that the *β* parameter is well reproduced with the fit function. These figures can be found in the Supplementary Fig. S1. In general, the simulated peaks are sensitive to the fit intervals for both *I*(*x*) and the full fit. A fit interval for *I*(*x*) that is slightly wider than the FWHM is needed in order to get a good fit for the simulations, probably since the Gaussian contribution here is small and thus *I*(*x*) contributed a larger portion of the peak.

### Fit to experimental data

**Fig. 2:**
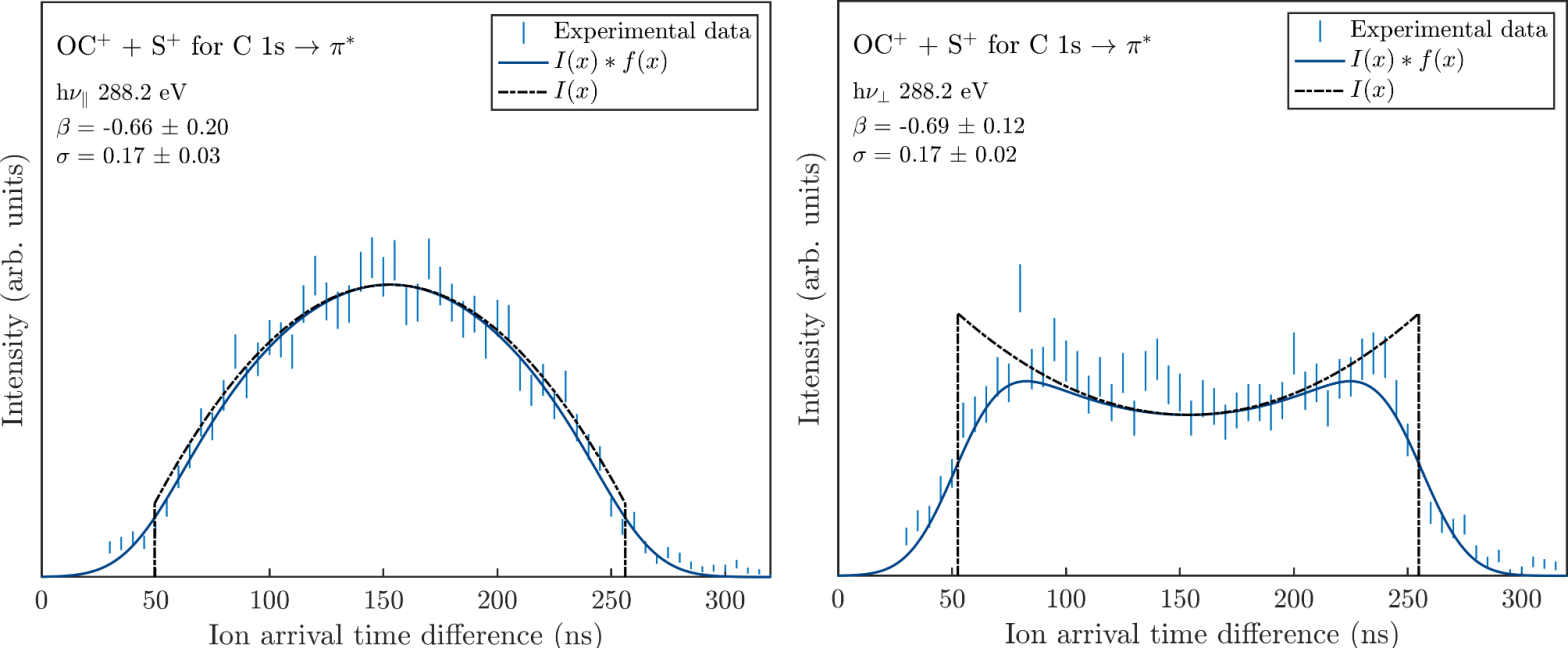
PIPICO peak shapes in blue error bars for the OC^+^ + S^+^ dissociation at 288.2 eV, for polarization parallel (h*ν*_∥_,) and perpendicular (h*ν*_⊥_) with the TOF axis. The blue solid line shows the fitted function, and the black dashed line is *I*(*x*) plotted with the obtained *β* parameter.

Moving on to experimental data, the same fit function is again used on experimental data alone. For OCS^2+^, data from experimental peak shapes are compared for h*ν*_∥_ and h*ν*_⊥_, where one dissociation channel at a specific photon energy should have the same anisoptropy parameter *β* regardless of the polarization direction. For the experimental peaks, the best fit is given when the interval for *I*(*x*) is approximately the FWHM of a slightly ∪-shaped peak (e.g. right panel of Fig. 2), and the full fit interval covers the entire peak range. Then, for the peaks that do not have a hollow shape, a similar interval (in TOF) is used. An example where the similar fit intervals are used for two different peak shapes can be seen in Fig. 2, for the OC^+^ + S^+^ dissociation at photon energy 288.2 eV (C 1s → *π*^∗^). The blue error bars represent the statistical uncertainty of the PIPICO shape, √*N*, and the solid blue line is the final fit. The fit parameters *β* and *σ* are seen in each panel (intensity parameter *a* omitted), with the corresponding 95% confidence intervals from the fit. Also, *I*(*x*) is shown in black using the *β* parameter obtained from the fit. The fit interval for *I*(*x*) corresponds to the FWHM of the right PIPICO peak shape and the interval for the full fit is well beyond the full peak. Both experimental peaks for the two polarizations in Fig. 2 give similar anisotropy parameter values of −0.67 ± 0.19. This value is well in line with Laksman et al.^12^, who obtained *β* = −0.67 ± 0.03, and also with Adachi et al.^4^ who reported −0.4, but without selection on two coincident ions (as pointed out by Laksman et al.^12^).

The OC^+^ + S^+^ PIPICO shape was also compared for two additional photon energies, 305 eV and 545 eV, where the fit function yields the same *β* parameter, within the error bar limits, for both polarizations. The corresponding figures can be found in the Supplementary Fig. S2. The standard deviation *σ* for the Gaussian distributions lie between 0.1 and 0.2 for all experimental peaks considered here. Since the Gaussian contribution is greater in the experimental peaks than the simulations, they are less sensitive to a poorly determined interval.

**Fig. 3:**
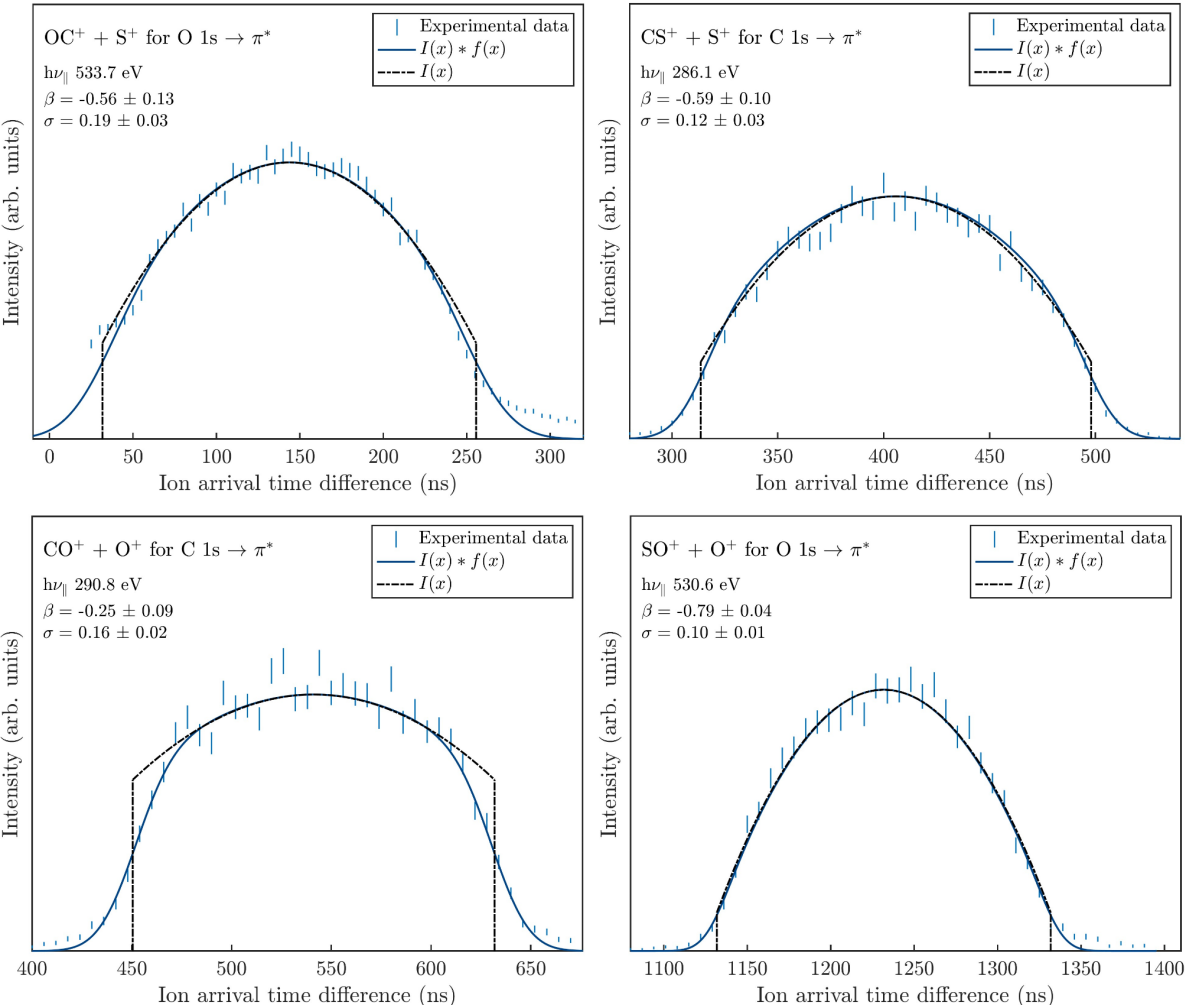
PIPICO peak shapes and anisotropy parameter fit for OCS (top left), CS_2_ (top right), CO_2_ (bottom left) and SO_2_ (bottom right), all with polarization along the direction of the flight tube axis.

Based on the same fit procedure, the anisoptropy parameter *β* has been determined from experimental PIPICO peaks and compared to previously published values for double ionization of OCS, CS_2_, CO_2_ and SO_2_, all with the light polarization parallel to the flight tube axis. The derived values of *β* are summarized in Table 1. For OCS, the *β* parameter at the O 1s → *π*^∗^ resonance was determined to *β* = −0.56 ± 0.13 (Fig. 3 top left), compared to Laksman et al.^12^ where *β* = −0.57 ± 0.03. Erman et al.^13^ reported *β* = −0.6 ± 0.1 for the CO^+^ fragment and −0.2 ± 0.1 for the S^+^ fragment, from PEPICO measurements, where the latter is possibly influenced by three-body processes.

**Table 1:** Anisotropy parameters *β* from the fit function on eperimental data in the middle column and literature values on the right. Adachi et al.^14^ do not provide error bars for their *β* values.

Dissociation pathway	Fit function	Literature values
OC^+^ + S^+^ (C 1s → π*)	−0.69 ± 0.19	−0.67 ± 0.03^12^, −0.4^14^
OC^+^ + S^+^ (O 1s → π*)	−0.56 ± 0.13	−0.57 ± 0.03^12^
CS^+^ + S^+^ (C 1s → π*)	−0.59 ± 0.10	−0.65 ± 0.1^15^, −0.6^14^
CO^+^ + O^+^ (C 1s → π*)	−0.25 ± 0.09	−0.48 ± 0.02^16^, −0.2^14^
SO^+^ + O^+^ (O 1s → π*)	−0.79 ± 0.04	−1^5^

For doubly ionized CS_2_, Adachi et al.^14^ determined the *β* value as −0.6 for the C 1s → *π*^∗^ resonance at 286.1 eV, compared to −0.59 ± 0.11 deduced from the top right panel of Fig. 3. These values confirm the previous *β* parameter obtained from PEPICO measurements by Karawajczyk et al.^15^. CO_2_ has a more flat topped PIPICO shape, with *β* = −0.25 ± 0.09 for C 1s → *π*^∗^ excitation at the photon energy of 290.8 eV. Again, Adachi et al.^14^ measured the anisotropy parameter over a range of energies, where for CO_2_ it varies between −0.04 and −0.4 over the *π*^∗^ resonance^14^. For comparison to our experimental data at the photon energy of 290.8 eV, their *β* is −0.2. Laksman et al.^16^ obtained −0.48 ± 0.02 for the *π*^∗^ resonance, not further specifying the exact photon energy used. Finally, in the bottom right panel of Fig. 3 the SO^+^ + O^+^ PIPICO peak shape at the photon energy of 530.6 eV is presented. The fit interval used for *I*(*x*) has been determined using the same peak, but at a different photon energy (535 eV) where the shape is slightly hollow and the fit interval can be determined more easily. The anisotropy parameter at the O 1s → *π*^∗^ resonance extracted from the bottom right panel of Fig. 3 is = −0.79 ± 0.04 which is in line with the value of −1 from the work of Gejo et al.^5^.

**Fig. 4:**
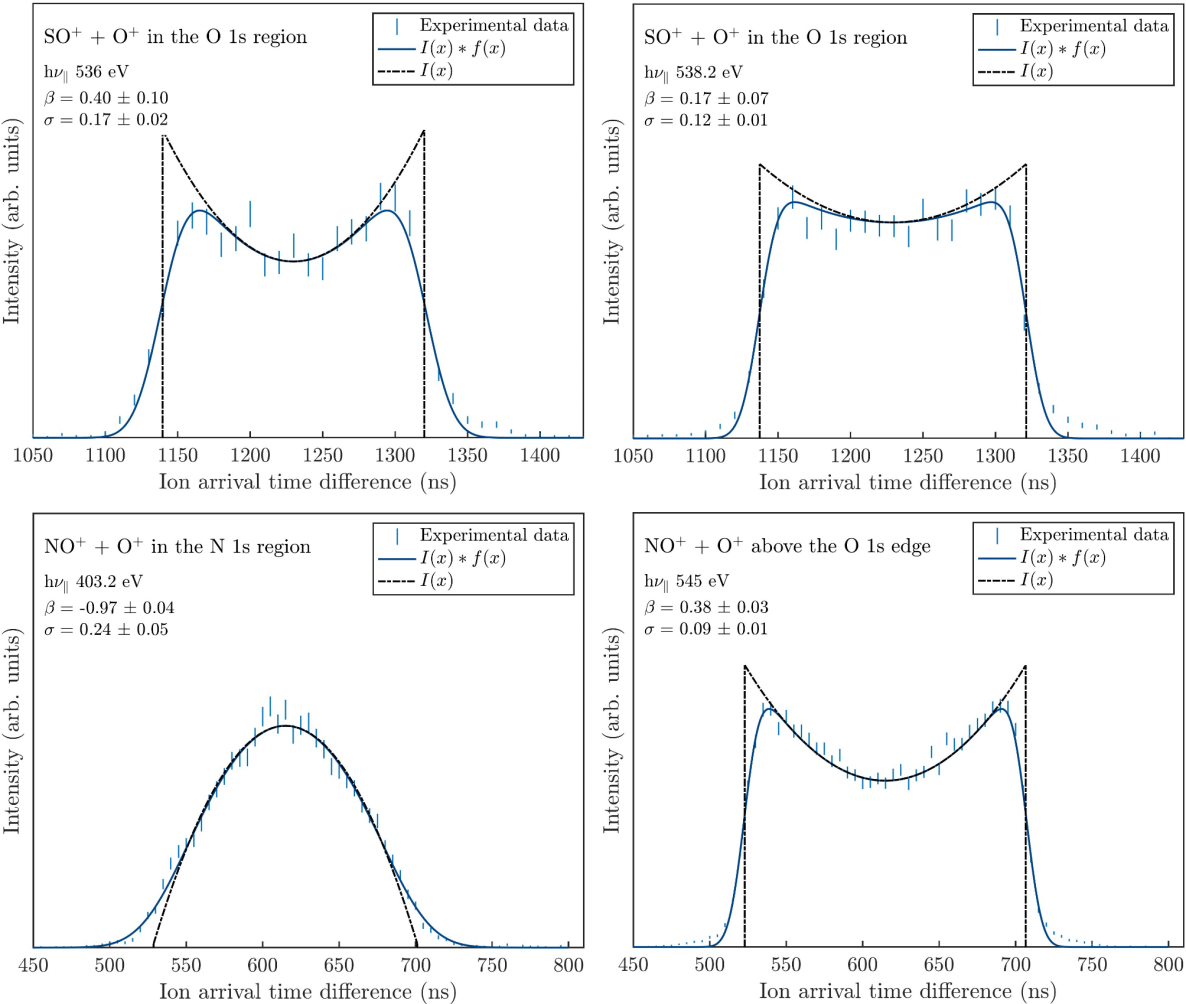
PIPICO peak shapes and anisotropy parameter fits for SO_2_ (top row) for two photon energies in the O 1s-edge region, and NO_2_ (bottom row) for one photon energy in the N 1s-edge region and one photon energy above the O 1s edge.

We have determined *β* parameters for four additional PIPICO peak shapes, shown in Fig. 4. The top left panel shows the SO^+^ + O^+^ peak shape at 536.0 eV, where the anisotropy parameter is found to be *β* = 0.40 ± 0.11. In the top right panel, the same peak is seen for a higher photon energy, 538.2 eV, where *β* = 0.17 ± 0.07. The bottom two panels show NO^+^ + O^+^ peak shapes from doubly ionized NO_2_. To the left, at 403.2 eV corresponding to the second N 1s resonance, *β* = −0.97 ± 0.04 is extracted, and to the right at 545 eV, above the O 1s edge, the anisotropy parameter is determined to *β* = 0.38 ± 0.03. For comparison, the angle-resolved ion yield spectra for these four peaks have been reported in the literature by Gejo et al.^5^, but without providing values for these *β* parameters. Apart from that, using the present method, we have also determined the anisotropy parameter *β* for the OC^+^ + S^+^ dissociation from OCS^2+^ for two more photon energies: at 305 eV, *β* = 0.12 ± 0.06 and at 545 eV *β* = 0.13 ± 0.22. The corresponding figure materials, for h*ν*_∥_ and h*ν*_⊥_, can be found in the Supplementary Fig. S2.

## Conclusions

A simple way to determine the anisoptropy parameter *β* for double photoionization from standard PIPICO peak shapes, where only one measurement angle and no comparison to simulated data is necessary, has been presented. The fit function we employed was tested on several simulated PIPICO peak shapes, to validate the use of the function for linearly polarized light for two perpendicular polarization directions. In addition, the use of the fit function was assessed with experimental PIPICO peak shapes alone, for dissociations with known *β* parameters and the *β* parameters obtained are found to be in good agreement with literature values. After validating our fit methodology, previously undetermined *β* parameters were evaluated for several dicationic two-body dissociation processes.

## Methods

### Experimental details

Ion-ion coincidence experiments on several molecular species were carried out in the photon energy range of 100–600 eV using linearly polarized light provided by beam line UE52-SGM of the BESSY II synchtrotron radiation storage ring at the Helmholtz-Zentrum für Materialien und Energie in Berlin. For the experiments, the storage ring was operated in single-bunch mode, where the time separation of the light pulses is 800.5 ns (1.25 MHz). This timing arrangement allowed us, using a mechanical chopper, to work with a light repetition rate in the range of 10–78 kHz^17^, which is much better suited for our experimental requirements. Time zero for ion time-of-flight measurements was set by a gate opened by a coincidence between the radio-frequency signal of the storage ring and a measured light pulse signal.

Wavelength selected synchrotron radiation intercepts the molecules from an effusive gas jet, injected into the interaction region via a hollow needle. Upon photoionization, the ions are extracted from the interaction region and then accelerated by two consecutive electric fields optimized for time-focusing conditions according to the focussing principles of Wiley-McLaren^8^. The ions are guided along a 0.12 m long drift tube, where the flight times are registered by a micro-channel plate detector coupled to a multi-hit analogue-to-digital converter system. The ions are identified by their flight times which are proportional to the square-root of their mass-to-charge ratio. The collection-detection efficiency is about 15% for mass 32 and the nominal mass resolution is ∆*m* ≈ *m*/40. To avoid ions originating from different ionization events being detected as artificial pairs, the detected ion rate was kept below 2% of the light repetition rate.

The photon energy provided by the beam line monochromator was calibrated by measuring ion count rates while scanning over known pre-edge inner-shell resonances of the target molecules. The radiation bandwidth was typically 50–100 meV.

### Theoretical model

The initial position of the molecular axis of the target species is uniformly distributed in three-dimensional space. Molecular orientations relative to the polarization of the light carry different absorption probabilities, depending on the photon energy and the molecular orbitals involved. The distribution of preferred molecular orientations is described by the anisotropy parameter *β* for a specific electronic transition. Values of *β* range from −1 to 2, with an intensity distribution given by

1 $$l\left( \theta \right)=\frac{1}{{4\pi }}\left( {1+\frac{\beta }{2}\left( {3{{\cos }^2}\left( \theta \right) - 1} \right)} \right)$$

where *θ* is the angle of the molecular axis relative to the direction of polarization^11^. In spherical coordinates, *θ* is the polar angle, *φ* the azimuthal angle and the *z*-axis is along the polarization direction.

In the present work, the light polarizations used were horizontal/parallel (h*ν*_∥_) and vertical/perpendicular (h*ν*_⊥_). The horizontal polarization was parallel to the TOF axis, i.e. towards the detector, whereas the vertical polarization was perpendicular to the TOF axis. In the laboratory frame, the form of the intensity distributions for a given photon energy are identical for the two polarizations, with a difference of a 90° rotation around the direction of light propagation.

**Fig. 5:**
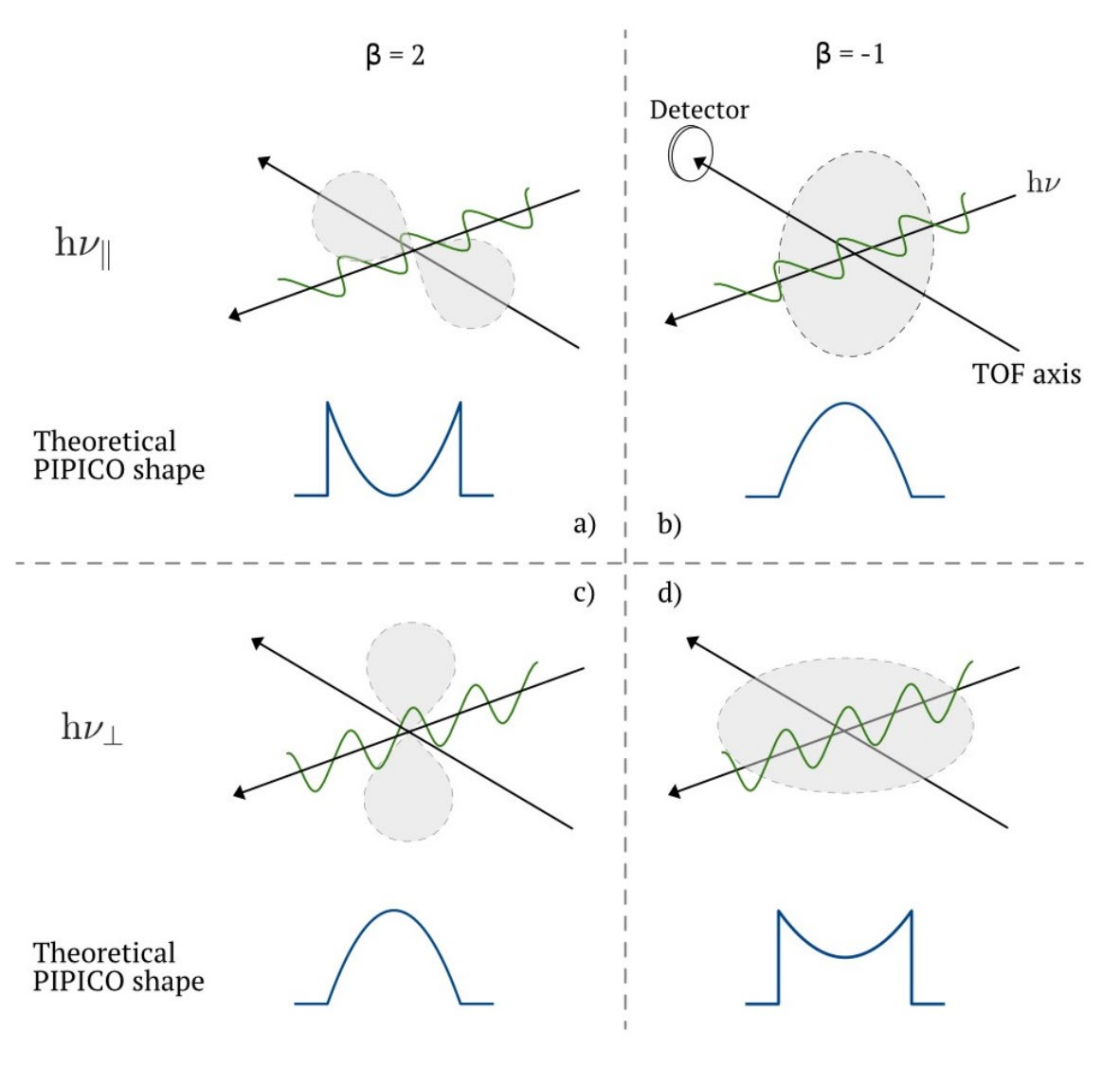
Schematic illustrating the propagation direction of the linearly polarized synchrotron radiation perpendicular to the axis of the ion time-of-flight (TOF) spectrometer. In (**a**) and (**b**), the radiation is polarized in the plane which contains the photon beam direction and the TOF axis, and in (**c**) and (**d**) the polarization is perpendicular to the TOF axis. In grey are the distributions of molecular orientations for *β* = 2 (left column) and *β* = −1 (right column), adapted from Zare^11^. The corresponding PIPICO shapes from *I*(*x*) are given in blue.

At *β* = −1, the intensity distribution is *I*(*θ*) ∝ sin^2^(*θ*). With arbitrary *φ*, the three-dimensional intensity distribution of preferred molecular orientations is a disc perpendicular to the polarization direction, as illustrated in Fig. 5b and d. At *β* = 2 the intensity distribution is *I*(*θ*) ∝ cos^2^(*θ*), i.e. molecular orientations in the direction of polarization, as illustrated in Fig. 5a and c. At *β* = 0 the intensity distribution is uniform (square “top hat” shape).

For two-dimensional momentum imaging measurements, this three-dimensional distribution *I*(*θ*) is projected onto the detector, giving an intensity distribution of *I*(*θ*)·sin(*θ*). This intensity distribution in the detector plane has been verified by empirical studies^6,12^. In our measurements, involving one-dimensional detection of the time of flight, the proper projection of the *I*(*θ*) distribution is realised with a non-linear variable change. The projection of the *I*(*θ*) distribution onto the time-of-flight axis does not directly obey the sin^2^(*θ*) or cos^2^(*θ*) shapes, as per *β* = −1 or 2 in *I*(*θ*) visualised as (*θ*, *I*(*θ*)). Instead, the correct empirical PIPICO-shapes are replicated by changing the x-axis: (cos(*θ*), *I*(*θ*)). By substituting *x* = cos(*θ*) into Eq. (1) the intensity form is.

$$l\left( x \right)=\frac{1}{{4\pi }}\left( {1+\frac{\beta }{2}\left( {3{x^2} - 1} \right)} \right)$$

where *x* ∈ [−1, 1], and *x* is assumed proportional to *t*_2_ − *t*_1_, the ion arrival time difference. Here, *I*(*x*) takes the forms simulated and described by Masuoka et al.^2^.

If the polarization is h*ν*_⊥_, the *I*(*θ*) distribution is rotated 90° around the light propagation direction, effectively rotating the distributions in Fig. 5a and b to c and d, respectively. According to Zare^11^, the difference between the two light polarizations is a scaling of *β* by a factor of −1/2 for h*ν*_⊥_. Defining *β*_⊥_ and *β*_∥_ for h*ν*_⊥_ and h*ν*_∥_, *β*_∥_ = −1/2 *β*_⊥_, which reproduces the correct PIPICO profiles for both polarizations. To understand this, a closer examination of the distributions in Fig. 5 is needed.

In Fig. 5, the two extremes where *β* = 2 and *β* = −1 are illustrated. For h*ν*_∥_, the two lobes of the intensity distribution in a), have forward and backward components along the TOF axis, with no perpendicular component, and will thus give a ∪-shaped PIPICO peak. For the disc in b), with only perpendicular components, the PIPICO peak will be ∩-shaped. If the polarization is instead h*ν*_⊥_, the distributions are rotated 90^◦^ around the light propagation direction. The two lobes, in c), are then perpendicular to the TOF axis. With no forward or backward components, the PIPICO peak will again be ∩-shaped, and is the same as in b). The disc shape in d), *β* = −1, will be in the plane of the TOF axis and the light propagation direction, giving forward and backward components as well as sideways, which gives a less hollow ∪-shape compared to the *β*_∥_ = 2 case in a), but the same PIPICO shape as for *β*_∥_ = 1/2 (not shown).

The shape of the PIPICO peak, *t*_2_ − *t*_1_, depends primarily on the KER and KERD together with the angular distribution relative to the light polarization. In addition, the peaks are affected by experimental factors, such as thermal width, light pulse duration, and spatial distribution. For accurate fitting of *t*_2_ − *t*_1_, and to account for all apparatus effects and the KER, it is necessary to convolute *I*(*x*) with a Gaussian, *f*(*x*). The Gaussian is given by.

$$f\left( x \right)=\frac{1}{{\sigma \sqrt {2\pi } }}\exp \left( {\frac{{{x^2}}}{{2{\sigma ^2}}}} \right)$$

centered at *x* = 0. An algebraic expression of the convolution *I*(*x*)∗ *f*(*x*), simplified in Mathematica^18^, is used as the fit function. This expression can be found in the Supplementary Materials. *β* is treated as a fit parameter, together with the standard deviation *σ* and an intensity scaling parameter *a*. In a first iteration the fit interval in nanoseconds for *I*(*x*), defined as [− 1, 1] for *x*, is also used as a fit parameter. Outside this interval *I*(*x*) is set to 0. With the interval parameter fixed from this first iteration, the fit is performed a second time, and the values of *β*, *σ* and *a* are determined. To also take the statistical uncertainty of the PIPICO peak into consideration, these two iterations of the fit are performed many times while randomizing the PIPICO peak intensity within the signal error bars. The final parameters presented are the mean values of the fit parameters from the many randomized peaks, with the 95% confidence interval.

**Fig. 6:**
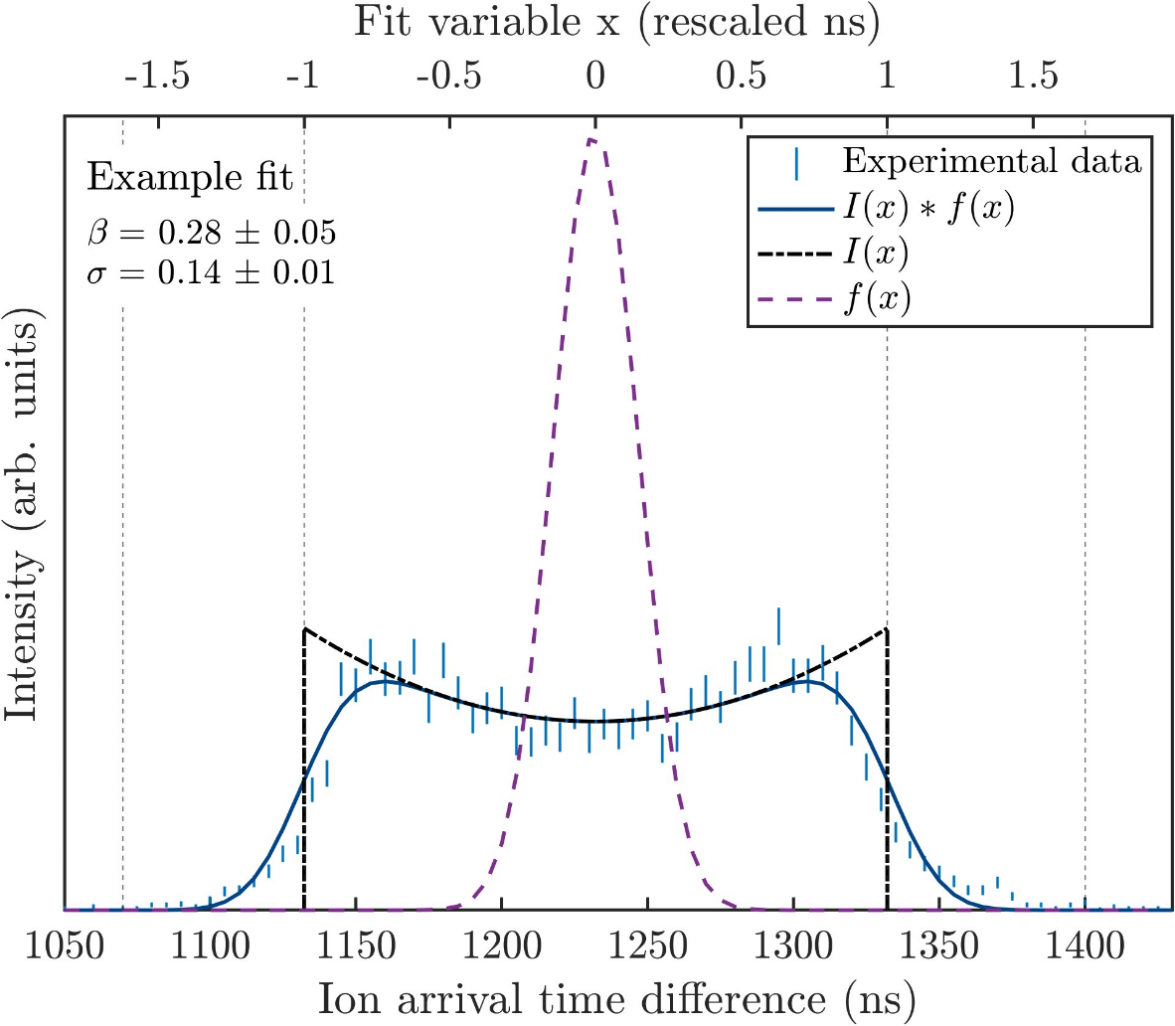
Example of the fit procedure, showing both the final fit in blue and the two convoluted functions *I*(*x*) and *f*(*x*) (black and purple), plotted using the obtained fit parameters, displayed in the top left corner. The upper horizontal axis represents the fit variable x, realized by determining the interval in nanoseconds (ns) where *I*(*x*) should be non-zero (innermost dashed grey lines), setting the edges to [− 1, 1] and rescaling the fit interval chosen in ns accordingly (outermost dashed grey lines).

An example of the fit to experimental data is shown in Fig. 6, where the fitted function *I*(*x*)∗ *f*(*x*) is seen in blue, and the two functions used in the convolution are plotted using the parameters *β* and *σ* obtained from the fit. The grey dashed lines show two different intervals, where the innermost is approximately the FWHM of the experimental peak, and, as referenced to the upper horizontal axis, this defines the [− 1, 1] range for *x*. This interval is used only to define where *I*(*x*) is nonzero (black dashed line). The outermost dashed lines show the interval covering the full peak, which is the actual fit interval used. In theory, this interval can be infinitely large, however it is only necessary to cover the full range of the peak.

### Simulations

Simulations of the ion flight paths upon two-body dissociation were performed in SIMION^19^, using a numerical replica of our apparatus, where the input ion distributions reflect the angular distributions for different anisotropy parameters *β*. All simulations consider two-body dissociations, with masses given by the dissociating ions, and the average KER is set to 5 eV (randomized from a normal distribution with mean 5 eV and a standard deviation of 1 eV) and the thermal width from T = 300 K.

The spatial distribution is randomized in spherical coordinates *r* = 1, *θ* (polar) and *φ* (azimuthal). For each ion pair, *φ* is randomized from [0, 2*π*], and *θ* is randomized from the probability distribution I(*θ*)·sin(*θ*), i.e. the anisotropic angular distribution as defined in Eq. (1) multiplied by a sine function to allow for the projection onto a two-dimensional surface. Then, for each ion pair, the molecular axis is given a direction based on the aforementioned distribution, and the molecular plane is rotated arbitrarily relative to the new direction. The difference between the two polarization directions is realized by a 90° rotation around the light propagation direction, defined by h*ν*_∥_ or h*ν*_⊥_. Hence, identical randomization procedures are performed for both polarization directions. The same fit function *I*(*x*) convoluted with a Gaussian is used for the simulated PIPICO peak shapes as for the experimental data, described above.

## Electronic supplementary material

Below is the link to the electronic supplementary material.

Supplementary Material 1

## Data Availability

The data sets generated during and/or analysed during the current study are available from the corresponding authors onreasonable request.
