# Supplementary material for: Anisotropy parameters from shapes of ion-ion correlation features of fragmenting molecules
Source: Sci Rep. 2024 Nov 20;14:28766. doi: 10.1038/s41598-024-80014-z (PMC11579029; doi:10.1038/s41598-024-80014-z)
Supplement: Supplementary file 1 — Supplementary Material 1 [file 41598_2024_80014_MOESM1_ESM.pdf]

Supplementary Materials
Anisotropy parameters from shapes of ion-ion correlation features of fragmenting molecules

Emelie Olsson[1], Måns Wallner[1], Richard J. Squibb[1], Veronica Ideböhn[1], Marco Parriani[1,2], Michael A. Parkes[3], Stephen D. Price[3], John H.D. Eland[4], and Raimund Feifel[1,*]

[1]University of Gothenburg, Department of Physics, Origovägen 6B, 412 58 Gothenburg, Sweden
[2]University of Perugia, Department of Civil and Environmental Engineering, Via G. Duranti 93, 06125 Perugia, Italy
[3]University College London, Department of Chemistry, 20 Gordon Street, WC1H 0AJ London, United Kingdom
[4]Oxford University, Department of Chemistry, Physical and Theoretical Chemistry Laboratory, South Parks Road, Oxford OX1 3QZ, United Kingdom
[*]raimund.feifel@physics.gu.se

Simulations - additional figures

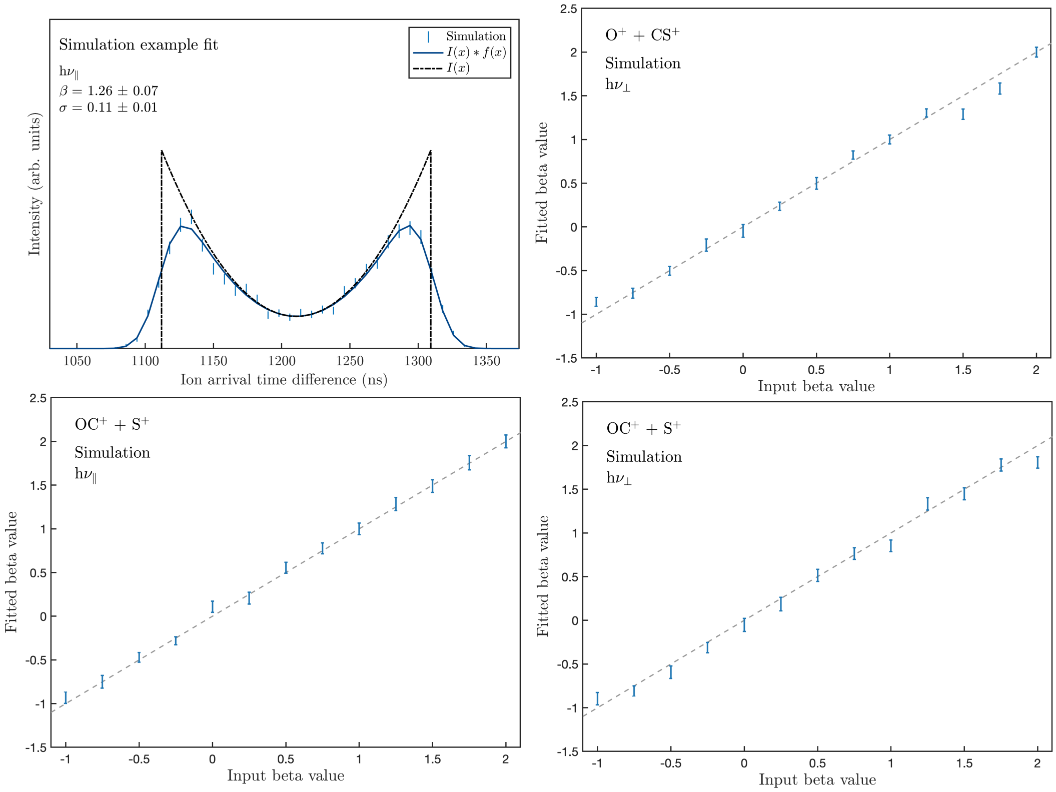

Figure S1. Example peak shape from the simulations (top left), for which the input β was 1.25. Comparison of the simulation input β and fitted β values for the $O^+ + CS^+$ dissociation for $h\nu_\perp$ (top right), and for the $OC^+ + S^+$ dissociation in the bottom panels, for $h\nu_\parallel$ (left) and $h\nu_\perp$ (right). The data point lengths represent 95 % confidence interval for β. For the bottom two panels the fit interval for $I(x)$ was limited to obtain good fits.

Experimental - additional figures

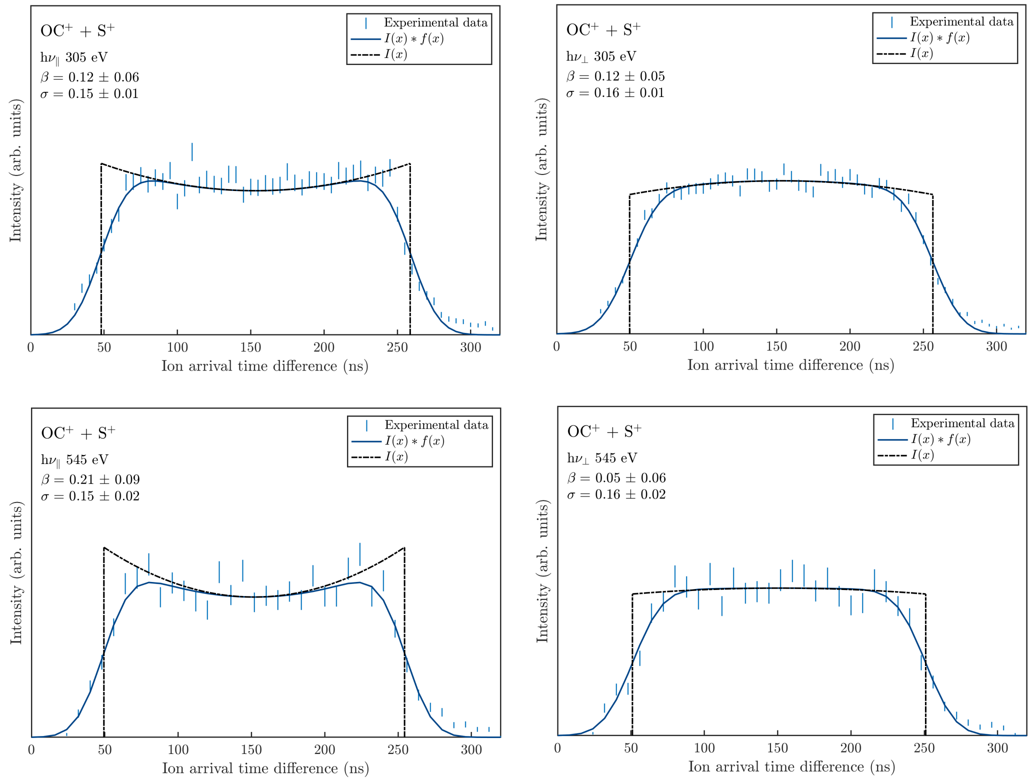

Figure S2. PIPICO peak shapes for the $OC^+ + S^+$ dissociation at 305.0 eV (top panels), and 545.0 eV (bottom panels), for h$\nu_{\parallel}$ (left) and h$\nu_{\perp}$ (right). For the bottom right panel, h$\nu_{\perp}$ at 545.0 eV, the fit interval has been set to be equivalent to the interval found for h$\nu_{\parallel}$ (bottom left). The blue solid line shows the fitted function, and the black dashed line is $I(x)$ plotted with the obtained β parameter. One dissociation channel at a specific photon energy should have the same β parameter regardless of polarization.

Algebraic expression for the convolution $\mathbf{I}(\mathbf{x}) * \mathbf{f}(\mathbf{x})$

The two functions convoluted are

$$I(x) = \frac{1}{4\pi}(1 + \frac{\beta}{2}(3x^2 - 1))$$

and

$$f(x) = \frac{1}{\sigma\sqrt{2\pi}} e^{\left(-\frac{x^2}{2\sigma^2}\right)},$$

where $I(x)$ is 0 outside $[-1,1]$ for x. Using Mathematica[1] and assuming only real values for x, the convolution $I(x) * f(x)$ is simplified, giving the following expression:

$$I(x) * f(x) = a\left(-3\sqrt{2}\sigma\beta e^{-\frac{(1+x)^2}{2\sigma^2}}\left(1 - x + e^{\frac{2x}{s^2}}(1+x)\right) + \sqrt{\pi}\left(2 + \beta(-1 + 3\sigma^2 + 3x^2)\right)\left(\operatorname{erf}\left(\frac{1+x}{\sqrt{2}\sigma}\right) - \operatorname{erf}\left(\frac{-1+x}{\sqrt{2}\sigma}\right)\right)\right)$$

which is further simplified by approximating the error functions with

$$\operatorname{erf}(x) \approx \tanh\left(\frac{2}{\sqrt{\pi}}\left(x + \frac{11}{123}x^3\right)\right),$$

again under the assumption of only real-valued x.

References

1. Inc., W. R. Mathematica, Version 14.0. Champaign, IL, 2024.
